# Electro-Therapeutics, Etc.

**Published:** 1876

**Authors:** Z. Collins McElroy

**Affiliations:** Zanesville, Ohio, Fellow of Zanesville Academy of Medicine, Member of Perry County Medical Society, Physician to the Home of the Friendless, etc., etc.


### Selections.

### ELECTRO-THERAPEUTICS—THE INSTRUMENTS USED—THEIR MATERIALS—THEIR SCOPE AND POWER, WITH CLINICAL ILLUSTRATION.

By Z. COLLINS McELROY, M.D., Zanesville, Ohio, Fellow of Zanesville Academy of Medicine, Member of Perry County Medical Society, Physician to the Home of the Friendless, etc., etc.

Reported at Session of Perry County Medical Society, October 14, 1875, held Junction City, O.

At your session in Roseville, last summer, I had the honor to read a paper on Electro-therapeutics, in which the different kinds of instruments in use were briefly described, and some illustrative cases were reported.

Since then I have given the subject such further attention as my time and opportunities have permitted, and it is my purpose to make another report to-day.

At the risk of some repetition—which I hope may not prove either unprofitable or uninteresting—I can best accomplish my ends by briefly calling your attention to the instruments again, for, it seems to me, that the first lesson of medical electrician is the scope, power, construction, and materials of the instruments used.

The most common instrument in the hands of the profession in the country is the magneto-electric machine, consisting of a permanent magnet, or magnets, sliding helix, and coils of insulated wire, set in operation by simply revolving the insulated coils past the poles, by a crank motion. For the reason that they are operated by a crank, I designate them the *Grinding Machines*.

The force generated by them, or to speak more correctly, made available by them, for medical use, is Magnetic, wholly and entirely; otherwise called the induced, or Faradic current.

Its physiological effect, ascertained alike by the simplest observation, as well as carefully conducted experiment, and its range of therapeutic application, is mainly limited to increasing muscular contractions. It is the "shocking" machine, *par excellence*, after the Leyden jar, of Franklinian electricity, or the electric current from friction. Remembering that the induced, Faradic, or magnetic current is confined in its effects, for the most part, to exciting increased activity of muscular contractions in living bodies, and limited in its results mainly to the parts to which it is directly applied, its range of application in the treatment of human sickness is seen to be very narrow. The tension of the current, or in other words, its velocity, is too high to be of much service alone in any case whatever. It must, therefore, take its place among scientific toys, or curiosities in our offices, useful mainly to astonish simple minded people.

The Electro-magnetic instruments of a quarter of a century ago, and still in use to a certain extent, differ from the grinding machines only in substituting for the permanent magnets, a weak galvanic battery, to do what the unfortunate PAYNE called "kicking up a fuss in the coils," and giving rise to the same magnetic current as the grinding machines, spending its force mainly in exciting muscular contractions at the points of application. In them there are generally two coils of insulated wire, of very different guage, or size and length. These coils are wound one over the other. The inner coil of rather coarse wire, and short length. Over this is a coil of fine wire of much greater length. At the point of union, between the secondary and primary, or coarse and fine wire coils, there is a vibrating armature, opening and closing the current with great rapidity, communicating a series of rapid shocks, varying in intensity with the position of the bundle of iron-wires, or soft iron rod, inside the inner coil.

Manufacturers have latterly placed on the markets, and thus in the hands of the profession, a style of electro-magnetic instruments under a new name, viz: Galvano-Faradic, claiming for them superiority over the old style electro-magnetic instruments. They differ, however, only in the greater number and length of coils, mechanical arrangements of parts, and means for applying currents to living bodies.

Partly on account of the resemblance between the sounds produced by the vibrating armature, and the wings of stinging insects, as well as from the sensations produced in living bodies in receiving their compound currents, I designate them "*Stinging Machines.*"

They all have one feature in common, and that is that they produce the induced, Faradic, or magnetic currents mainly; and as has already been stated, experiments have shown that these mainly influence muscular contractions. They have one common merit, and a very great one it is, all things considered, and that is, they are portable. Some of them are elegant. It is to be sincerely regretted that they are not adapted to the treatment of a wider range of human ailments.

It is perhaps known to you all that there are but two primary currents—viz: the direct, or galvanic current, derived from the immersion of suitable plates in an acid, or saline bath, and the induced, Faradic, or Magnetic current, which is obtained by passing the direct current through a coil of insulated wire, with a bundle of iron wires, or soft iron rod, inside of the coil, which unites with the direct current, and greatly alters and intensifies its effects in living bodies.

The direct, or simple galvanic current, is the current of electrolysis, or chemical decomposition of compound bodies, inorganic, or organic. It varies in intensity with the extent of surface of zinc and copper, or other materials, exposed to the action of the acid, or saline bath, or contact. The greater the number of small plates, the greater the tension, or intensity of the current, up to the point of what is called the galvanic cautery, so useful in certain surgical proceedings now a days. From a single pair of small plates, say 4½ or 5 inches long, by 1½ to 1¾ inches wide, up to any number of such pairs, the quality and character of the current is the same; its only variation is in its quantity and velocity. The current varies with the materials in the plates, to some extent, in quality and velocity, at least in its therapeutical effects. But no matter how produced, it is the current of electrolysis, or electro-chemical decomposition of compound bodies. In other words, the current quickening the pace of molecular work in living bodies, notably in the interest of waste of existing tissue, though operating also in the interest of repair of wasting tissues, as it quickens the speed of all molecular work.

The induced, magnetic, or Faradic current, from one, or any number of coils, possesses the same character, and has the same influence, therapeutically, viz: increasing muscular contractions. This is made evident when the current is opened and closed in more or less rapid succession. If somewhat powerful, and the current is opened and closed rapidly, its influence on muscular activity amounts to clonic spasm, in addition to a series of disagreeable shocks.

The galvanic current is, like all effective work in nature, silent in its operation, giving rise to no unpleasant sensations, no matter how slowly or rapidly the current is opened or closed, except when the surface of application is too small for the quantity, or intensity of current, when there is a burning sensation on the skin, or other surface to which it is applied. This may, as it has in a few cases in my hands, readily produce vesication, and it does it very quickly, too, when the current is strong.

The united currents, as one or the other preponderate in the compound current, produce the effects of both in the ratio of their combinations, though the induced current cannot exist without the Galvanic. The Galvanic is the independent current.

In all the portable instruments there is just sufficient Galvanic action to give rise to the induced, or Faradic current, or currents, as the coil is simple, or complex; that is, Galvanism is minus; magnetism, or induced current, or currents, plus the very opposite of what it should be, and must be, to be successful in the hands of the many. The range of application for them is much wider than with the grinding machines, but they, one and all, as offered by different manufacturers and inventors, fall short, very far short, of equipping the general practitioner for the practice of what is called electro-therapeutics, if successful treatment is aimed at in their use. In a word, the apparatus for the general practitioner has not yet been offered in our markets. I think it not improbable that Galvanic apparatus may yet be offered so cheaply, that when needed for persons who cannot, or prefer not to visit physicians offices, they will be bought by patients, just as they now get prescriptions filled, the practitioner carrying with him the coils and means for its application only. It ought to be so now, for in a simple Galvanic battery there are no expensive materials, and no necessity for expensive mechanical work on them. The cup in use by the Western Union Telegraph Company, all over the United States, where their lines extend—the Callaud—can be delivered almost anywhere in the United States for less than two dollars per cup, including the necessary connections between them ; and four to six cups form a sufficiently powerful battery for all medical purposes, though not for the Galvano cautery. It is emphatically the "ever-ready" battery, needing less attention, perhaps, than any other now known. They work continuously in telegraph offices about one year before a renewal of their essential parts of copper and zinc becomes necessary. All that they require is the occasional removal of the upper stratum of water charged with zinc sulphate, the addition of fresh water, and copper sulphate as needed. In them the copper and zinc are suspended in one glass vessel holding about a gallon. The zinc is in the form of a wheel, and is hung near the top of the cup. The copper is in three thin slips, rivetted together in the center, and spread out like leaves of a book, and lies on the bottom of the vessel, having an insulated wire running up the side of the vessel to connect with the zinc of the next cup. In an office or private dwelling, such a battery may be placed in any out of way place, on a shelf, in a cupboard, or cellar, or garret. The necessary insulated wire, screw clamps, etc., to connect the battery with a table, or stand, cannot add much to the expense. A suitable coil and means for practical application can hardly cost more than the battery, so that a really efficient battery, and means for its application can be had for from twenty to twenty-five dollars. The requisite skill for its application can only be had from physicians. I have dwelt on the Callaud battery somewhat, because I think it, or possibly some cheaper and better battery which may be devised, is infinitely better than any portable battery yet made for medical purposes. It supplies the galvanic current in much greater volume than any portable apparatus can, and it is with this that the most good can be done medically. All the portable apparatus supply only the Galvanic current needful for the production of the magnetic current, or currents, in the coils. The physician needs, not the magnetic, but the current of electro-chemical activity, in excess, in the bulk of cases proper for its use. And this is the galvanic current. There is magnetic current sufficient to rouse up muscular activity in an ordinary telegraph sounder. And this form of coil enables the operator at any time to assure himself of the actual working of the battery, and the character of the click is a very good index of the strength of the combined Galvano-magnetic current. I object to the portable apparatus in use, also, on account of the rapidity with which the current is broken and closed. Unless very weak, it is excessively disagreeable, and it does not seem possible amidst such a hub-bub, to and fro —current—that as much good can be obtained in any case, as when the current is slowly interrupted, or, in some cases, not interrupted at all. The impression gains on me all the time that the disappointments met by the general practitioner in the use of electricity, are largely due to the defective apparatus in the really handsome portable form in which it is supplied to him by dealers. They doubtless do good in a certain range of cases, but their general applicability falls far below that which I have endeavored to describe. Aside from the form of any apparatus, is the liability of all of them to get out of order, except the grinding—this seldom fails, if the crank is made to revolve. Some mechanical skill is needed to keep any of them in working order, for the most apparently trifling circumstances prevents their working.

Therapeutically, the Galvanic and induced currents, singly or combined, interrupted aud continuous, do certain things in living bodies which it is not now possible to accomplish with known modes of "stored up force" in drugs or medicines. And the converse is also true, that certain things can be, and are done, by "stored up force" in medicinal substances, which it is not now possible to do with Galvanism or Magnetism. What Galvanism and Magnetism do, I think of as quickening the pace of molecular work; and yet that concept does not cover the ground. To complete the conception must be added the mod ification of the mode of molecular works.

As illustrating one of the results of the latter kind, I submit the history of a case, kindly furnished me by Dr. J. F. Kennedy, of this city, at whose request I made the electrical application, and to one of his patients.

"George T., aged 10, obtained the following history from his mother. George went to visit an invalid aunt living in the country, about the 15th of May last, at that time was in his usual perfect health, weighing 89 pounds. He remained in the room occupied by his aunt almost constantly during his stay in the country, about eight weeks. His aunt died of consumption, and George returned home. I was called to visit him, 24th of July, 1875, and found him in the following condition: pain in the chest; feet and ankles swollen, though only slightly; dry, hacking cough; skin moist through the day, and sweats profusely at night; tongue slightly coated; appetite good; bowels constipated; pulse 130; sleeps almost constantly—scarcely remains awake long enough to eat his meals; his mother thinks he has slept twenty out of every twenty-four hours since his return home; he is greatly reduced in flesh. Prescribed Calomel, grs. iij, divided into three powders, to take one every three hours till his bowels move. After his bowels moved, gave him Cod Oil, with Citrate Iron and Quinine, together with all the native wine he would take. Had his body sponged every night with diluted alcohol. This line of treatment was continued until 13th of August, without any improvement. Friday, 13th of August, took him to Dr. McElroy's office.

| | | | |
|---|---|---|---|
| "Weight 14th | of August, | 59 | pounds. |
| " 21st | " | $60\frac{1}{2}$ | " |
| " 28th | " | 63 | " |
| " 4th | of Sept., | 66 | " |

"From this date he continued to improve rapidly, and was dismissed 27th September, well."

The day the lad was brought to my office, he had a temperature of a 103° F. Pulse 130. Skin dry and hot. He was stripped naked, except pants, feet placed in warm water, and received the Galvano—Faradic current in which the Galvanic action was plus, or in excess largely of the induced, or Faradic action. The last carbon plate of the battery (10 cups, plates, $1\frac{3}{4}$x5 inches, immersed in saturated solution sulphate copper, $2\frac{1}{2}$ inches), was connected with the foot bath. The zinc, after passing through the coil, was, by the usual flexible insulated cord, connected with a small piece of thin flexible brass, 2x$1\frac{1}{4}$ inches, wrapped in a small white flannel napkin, wet with plain water. This was applied to his head, back of neck, back and front of chest, and abdomen, and along the spine, a part of the time through my own person. He was in the circuit about 12 minutes. The current was through a short coil, of moderately fine wire, and was not strong enough to be unpleasant, or alarm him when it was interrupted, which was done frequently, or from five to ten times per minute. At the time of making the application to the lad, I confess, I did not expect him to derive much benefit from it. I made it as much out of courtesy to Dr. Kennedy as anything else. The boy seemed to enjoy the thing while he was in it, though he was somewhat alarmed at being stripped. Still, I made the thing as jolly as I could, and kept him amused and smiling much of the time. And he seemed to enjoy it. He was to return for a second application in three days, but he never came back for another. Dr. Kennedy tells, he called for him at the appointed time, but found him unwilling to come. Said he had seen lightning, and felt queer all over when I applied it to him the first time, and he was afraid to go again. And as he seemed so unwilling the matter was postponed, and as his condition from day to day was so satisfactory, the subject was not mentioned to him again. Dr. Kennedy tells, that the sweating was much less the first night after the electrical application, and ceased after the third; that his pulse came down much lower than it had been; that he did not sleep nearly so much; did not have any pain in the chest; his appetite became better from day to day, and that, in fact, the electrical application made a complete and total change in the lad. If it was not that, there was, to say the least of it, a remarkable coincidence between the electrical application and the turning point toward recovery. The interest of the case hinges largely on the view taken of its pathology. It had some features in common with what is called continued fever, though far from being a typical case. Some of its features are not often present in what we call typhoid fever. The somnolency and sweating were features pointing in another direction, as was also the cough, constipation, and condition of nutrition. Without the close association with his dying aunt, during two months, it would probably be regarded as an anomolous case of continued fever. But his close connection with his aunt during the last months of her consumptive life, points, it seems to me, unmistakably, to the real pathology of the case. Then his subsequent progress, until he came under the care of Dr. Kennedy, seemiugly not changed by his professonal interference, certainly not for the better, until after the electrical application. The state of the circulation, respiration and the temperature, on the day he received the electrical application, was such as might be expected on the twentieth day of fever, providing it had been seen by Dr. Kennedy on its initial day. But Dr. Kennedy did not see it at its commencement, in all probability. Two weeks had elapsed since his return from the country, when first seen by Dr. Kennedy. His loss of weight was large, nearly thirty pounds, or one-third of his weight before going to the country. It seems to me the conclusion that he had, so to speak, became infected by his dying aunt, cannot be resisted. But I will not dwell on these points, seeing that the processes taking place in the bodies of those passing through the phenomena of typhoid fever, and tuberculosis, are identical in kind, but not in mood. Both wasting existing flesh by the process we call fever. But with this difference in modes. The wasting by that of typhoid fever, having the possibility of ultimate reconstruction of the structures in normal molecular forms; while with tuberculosis it is wasting with no probability of repair from new material, so as to regain health. The one tends inevitably to dissolution; the other to recovery. To my mind it is clear that the lad had what we call tuberculosis.

With this conclusion the importance of the results of the solitary electrical application assume very great importance. Could the tide of life, so to speak, in the lad have been turned by any other known means so suddenly and so sharply? Possibly so; possibly not. Probably not. The electrical application established at once the process of repair, for at the end of a week he has gained one and one-half pounds. At the end of two weeks, four pounds; and the third seven pounds, or almost one-eighth of his weight in twenty-one days; a very remarkable gain.

I can even go farther, and explain more or less satisfactorily to my own mind how it was done. The tendency of effete products of the combustion, or waste of tissue, is to take crystaline forms, strikingly illustrated in calculous affections, tubercles, etc., so called. The condition for this solution are absent often times; and the crystalisation is interstitial. That was probably the case with the body of the lad. The water, holding a certain quantity of saline matter in solution, which would probably have been sufficient for this purpose in a simple fever, escapes by the skin, leaving the crystals intact, and even enlarging each day. The electrical application, it must be remembered, was plus Galvanic—electrolytic—the current of decomposition of complex substances, with just sufficient magnetic action—the muscular contractor—to set these crystals in motion, to decomposition, and solution, and removal. The leaking at the skin ceases at once, the water hitherto escaping there, turned into other channels, and to perform its proper and important functions in the lad's body. Then repair becomes possible, and commences at once, and in twenty days he gains nearly one-eighth of his total weight. But this does not represent all of his gain. The waste, or removal of spoiled tissure, by the fever process, still proceeded, but this is made good, and the gain of seven pounds in total, weight, represents so much additional.

The lad speedily regained his lost health, and, under the circumstances, could hardly have done otherwise. It does seem to me that the galvano-faradic action in this case was the direct and principal means of changing, in toto, the processes taking place in the lad's body, and there is not much probability that anything else could have done so, so promptly and effectually. I think that had the current been from one of the misnamed Galvano-faradic machines—in other words, mainly magnetic—the result would have been negative.

I confess I have learned something from this incident, which I hope to turn to the profit of other patients in the future.

1. From a rather close and extended study of the principles involved in the production and application of the galvanic and faradic currents, clinically, I am forced to conclude that there is no form of portable apparatus offered in the market which places at the disposal of the actual, working practitioner, the quality of current needed in the successful management of the great bulk of cases coming under his care to which it is applicable, or in which electro-therapy is indicated. What he wants is the current of electrolysis, the current of chemical decomposition of compound bodies; for it will be necessary, in the main, to hasten the removal of effete matter—matter not available for the purpose of life—as well as the decay of tissue which has lost its dynamic capacities in living bodies, which is the real pathology of a large proportion of chronic cases which are or can be benefited by electricity.

2. That any really effective apparatus must, in the nature of things, be more or less complex, requiring a certain amount of mechanical skill to keep in order.

3. That application through the hands and arms, by means of the tin or other metallic handles, always a prominent feature of the equipment of a portable machine, is, in the great bulk of cases, entirely useless.

4. That water is the electrode better than all others, except for cautery purposes, or application to extremely small surfaces.

That any piece of bright and better flexible sheet metal, wrapped in wet cloth, preferably flannel, though cotton, linen, or silk answers very well, forms an electrode of the best possible character, better than metals or sponges.

5. That the feet of the patient, as a general thing, ought to be immersed in warm water, or placed on a wet napkin, for general applications, passing current from back of neck along spine; over chest, back and front, dwelling on parts tender or painful. The ceuben, or copper pole, at feet, zinc to superior parts.

6. That applications about the head must be cautiously made, as it is much more sensitive than any other part of the body, except the hands.

7. That applications altogether local are useful and necessary, but succeed better after general application than alone or singly.

8. That the class of cases most promising are those in which ordinary medicines have failed, and continue to fail, when given alone. But that the clinical case here reported will warrant its further application in acute stages of some kinds of sickness. Properly given it seems to me they can do no harm.

9. That no application, whether general, or local, should give rise to actual pain, or be unpleasant, if it is expected to do any good.

10. Electricity can be made to do the work of blisters and other counter irritants, in some cases, more pleasantly and speedily than counter irritants themselves, but does not supersede their use by any means.

11. That electro-therapy does not take the place or render unnecessary proper medicines, when indicated. It merely supplements their action and makes their work more efficient.

12. For, there are but two possible effects which galvanic and magnetic action can have on living tissues, viz.: hurrying up and modifying the speed and mode of molecular work. No material is supplied, only motion to existing material. As medicines represent "stored up force," so I think of electricity as naked force—force disconnected from matter as it enters a living body.

